# Macrolides at Clinically-Relevant Concentrations May Induce Biofilm Formation in Macrolide-Resistant *Staphylococcus aureus*

**DOI:** 10.3390/antibiotics12020187

**Published:** 2023-01-17

**Authors:** Carlos F. Amábile-Cuevas

**Affiliations:** Fundacion Lusara, Mexico City 08810, Mexico; carlos.amabile@lusara.org; Tel.: +52-(555)-219-5855

**Keywords:** Biofilm, *S. aureus*, macrolides, erythromycin, clarithromycin, azithromycin

## Abstract

Macrolides inhibit biofilm formation in several Gram-negative, intrinsically-resistant bacterial species. However, the effect of macrolides upon biofilm formation by susceptible Gram-positive bacteria has been much less explored as such concentrations also inhibit cell growth. To circumvent this problem, the effect of macrolides (erythromycin, clarithromycin and azithromycin) at 0.5–2 µg/mL, upon biofilm formation, was explored on macrolide-resistant *Staphylococcus aureus* isolates, using the crystal violet assay with 96-well plates. Early (4 h) biofilm formation by strains having constitutive target-modification resistance was consistently induced by all macrolides but not in azithromycin-treated cells in longer (8 and 12 h) incubation. In inducible-resistance isolates, early biofilm formation was enhanced by some macrolide treatments, compared to similar cell growth in the absence of antibiotics; but the typical decay of biofilms at longer incubation appeared prematurely in macrolide-treated cultures. Biofilm formation in an efflux-mediated resistant isolate was not affected by macrolides. These results indicate that macrolides induce the formation of biofilm by resistant *S. aureus* isolates, especially during the early stages. This suggests that the empirical use of macrolides against infections caused by resistant *S. aureus* strains could not only result in clinical failure but even in the enhancement of biofilms, making further treatment difficult.

## 1. Introduction

Macrolide antibiotics are known to exert a number of effects aside of the mere inhibition of protein synthesis by bacterial ribosomes. In patients, they reportedly have pro-kinetic activity in the gastrointestinal tract, as well as anti-inflammatory (or “immunomodulatory”) properties [1]. In intrinsically resistant bacteria, such as *Pseudomonas aeruginosa* (e.g., [2]), *Klebsiella pneumoniae* [3] or *Acinetobacter baumannii* [4], macrolides are capable of inhibiting biofilm formation. The basis of the anti-biofilm effect of macrolides has not been completely elucidated: for instance, azithromycin seems to block quorum signaling in *P. aeruginosa* [5], and has been reported to also inhibit alginate production [1], an important component of pseudomonal biofilms. In any case, since the effect is achieved at sub-inhibitory concentrations, it is assumed to be of non-ribosomal nature.

Although the pharmacokinetics of macrolides differ widely, maximum plasma concentrations (C_max_) of erythromycin (ERY), clarithromycin (CLA) and azithromycin (AZI) after oral administration is within the 0.5–4 µg/mL range, depending on the drug, dose and formulation [6,7,8]. Exploring the effect of macrolides at clinically-relevant concentrations upon biofilm formation by intrinsically-resistant bacteria can be done with ease, as such concentrations are not inhibitory for these microorganisms. For instance, AZI retards biofilm formation in *P. aeruginosa* at 2 µg/mL, but the minimal inhibitory concentration (MIC) of the strains used was 128 µg/mL [9]. However, in susceptible organisms, macrolide concentrations that are achievable clinically are enough to inhibit bacterial growth, making it difficult to assess the non-ribosomal effects, such as those upon biofilm formation. Susceptible *S. aureus* isolates, for instance, have MICs in the range of 0.032–0.125 µg/mL (resistance breakpoint is ≥8 µg/mL for these macrolides, while susceptibility breakpoints are ≤0.5 µg/mL for ERY, and ≤2 µg/mL for CLA and AZI). For this reason, macrolide-resistant *S. aureus* isolates were selected for this study on the effects of macrolides upon biofilm formation.

Previous reports have shown the anti-biofilm activity of macrolides upon *S. aureus*, but are still controversial [10]. Most of these studies have been carried out with extended, ≥24-h incubations resulting in “mature” biofilms [11]. A purported anti-biofilm effect of CLA was deemed of little clinical relevance in treating staphylococcal infections based on observations in a rat model [10]. On the other hand, macrolides at 1/4xMIC (4–32 µg/mL) enhance the biofilm formation of macrolide-resistant isolates of *S. epidermidis* in 20% [12]. In addition to neglecting the dynamics of biofilm formation by only measuring the effects at a late-stage, few studies characterize the activity of macrolides depending on the mechanism of resistance. Staphylococci can gain resistance to macrolide drugs through the acquisition of *erm* (*e*rythromycin *r*ibosome *m*ethylase) genes that confer resistance to macrolides, lincosamides and streptogramin B (named MLS_B_ phenotype, which in turn can be either inducible, iMLS_B_, or constitutive, cMLS_B_); or of macrolide/macrolide-streptogramin B efflux genes (*mef* or *msrA*, respectively). Other macrolide resistance mechanisms, such as drug inactivation, are rather rare [13]. Each mechanism differently affects the growth under macrolide presence and/or the intracellular concentration of the antibiotic. Here, I show that macrolides appear to induce biofilm formation in MLS_B_ isolates, especially during the early stages of development; while this was observed in an in vitro model of biofilm formation, if similar effects occur in clinical conditions, it could hamper antimicrobial treatment if macrolides are empirically used against resistant strains.

## 2. Results

### 2.1. Effect of Macrolides upon Biofilm Formation by cMLS_B_ Strains

**Figure 1:**
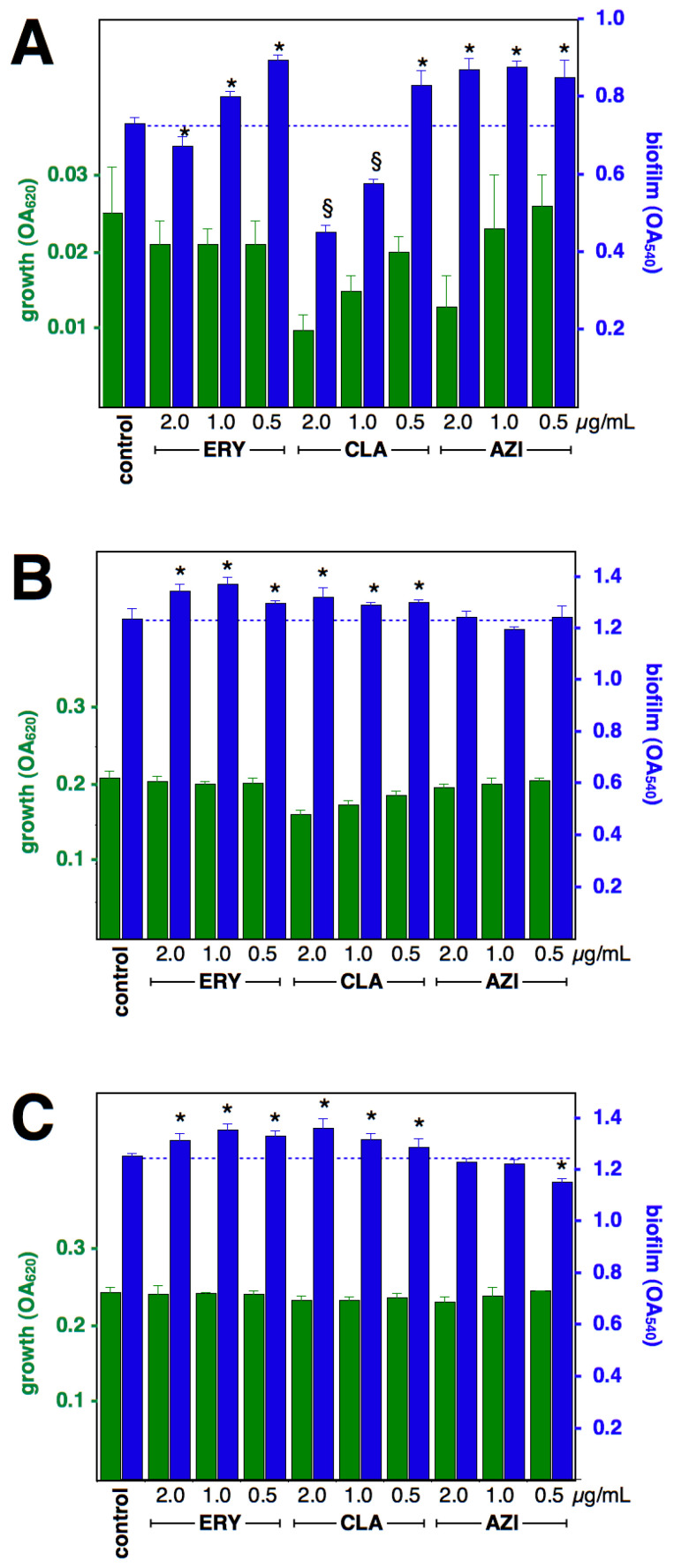
Effect of macrolides upon biofilm formation by *S. aureus* strain RN11 (constitutive MLS_B_). Growth (green) and biofilm formation (blue), at 4 h (**A**), 8 h (**B**) and 12 h (**C**), shown as OA at 620 and 540 nm, respectively (means and standard deviations of four independent experiments). * *p* < 0.05 by Student *t*-test against control. Growth was significantly (*p* < 0.05) reduced by AZI 2 µg/mL/4 h and CLA 1 and 2 µg/mL/8 h, yet biofilm formation was significantly larger than control. § not statistically evaluated, due to diminished growth.

**Figure 2:**
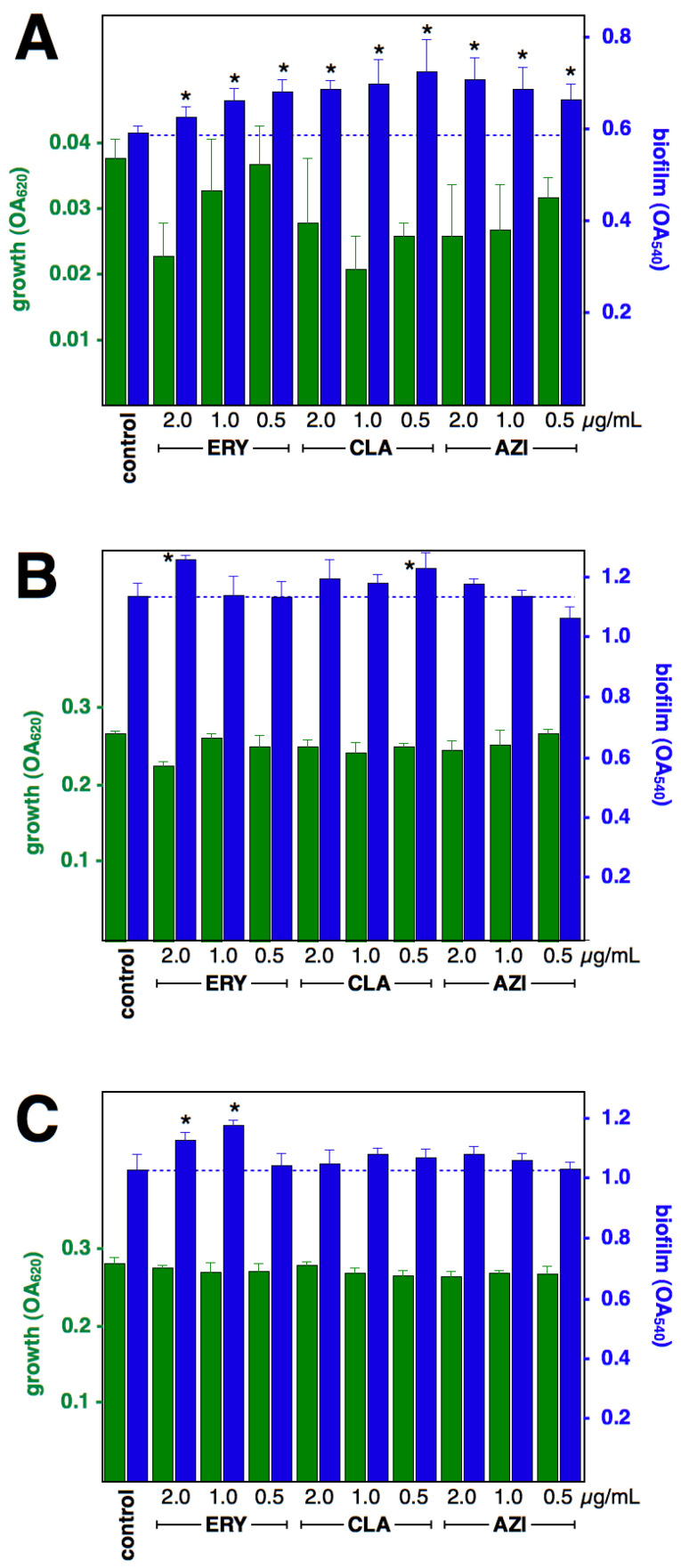
Effect of macrolides upon biofilm formation by *S. aureus* strain c2 (constitutive MLS_B_). Growth (green) and biofilm formation (blue), at 4 h (**A**), 8 h (**B**) and 12 h (**C**), shown as OA at 620 and 540 nm, respectively (means and standard deviations of four independent experiments). * *p* < 0.05 by Student *t*-test against control. Growth was significantly (*p* < 0.05) reduced by ERY 2 µg/mL and CLA and AZI at 4-h incubations and by ERY 2 µg/mL/8 h, yet biofilm formation was significantly larger than control.

The effect of macrolides at 0.5, 1 and 2 µg/mL upon the growth of cMLS_B_ isolates after 4-, 8-, or 12-h incubation was mostly negligible (Figure 1 and Figure 2); therefore, a direct comparison of biofilm formation can be made. The effect upon biofilm formation differs between drugs and between strains; all three macrolides significantly induced early formation (4 h) at all three concentrations tested (except for ERY 2 µg/mL upon strain RN11, which resulted in a significant reduction of biofilm formation; CLA 1 and 2 µg/mL inhibited cell growth, hence the comparison of biofilm formation against the antibiotic-free control was not possible). After 8-h incubation, ERY and CLA treatments were still inducing increased biofilm formation in strain RN11; but only 2 µg/mL ERY and 0.5 µg/mL CLA resulted in increased biofilm formation in strain c2. After 12-h incubation, strain RN11 showed increased biofilm formation when under ERY and CLA treatments, but 0.5 µg/mL AZI resulted in a reduction in biofilm. In strain c2, only ERY 1 and 2 µg/mL resulted in increased biofilm formation, while the rest of the treatments did not change the formation of biofilm.

### 2.2. Effect of Macrolides upon Biofilm Formation by iMLS_B_ Strains

**Figure 3:**
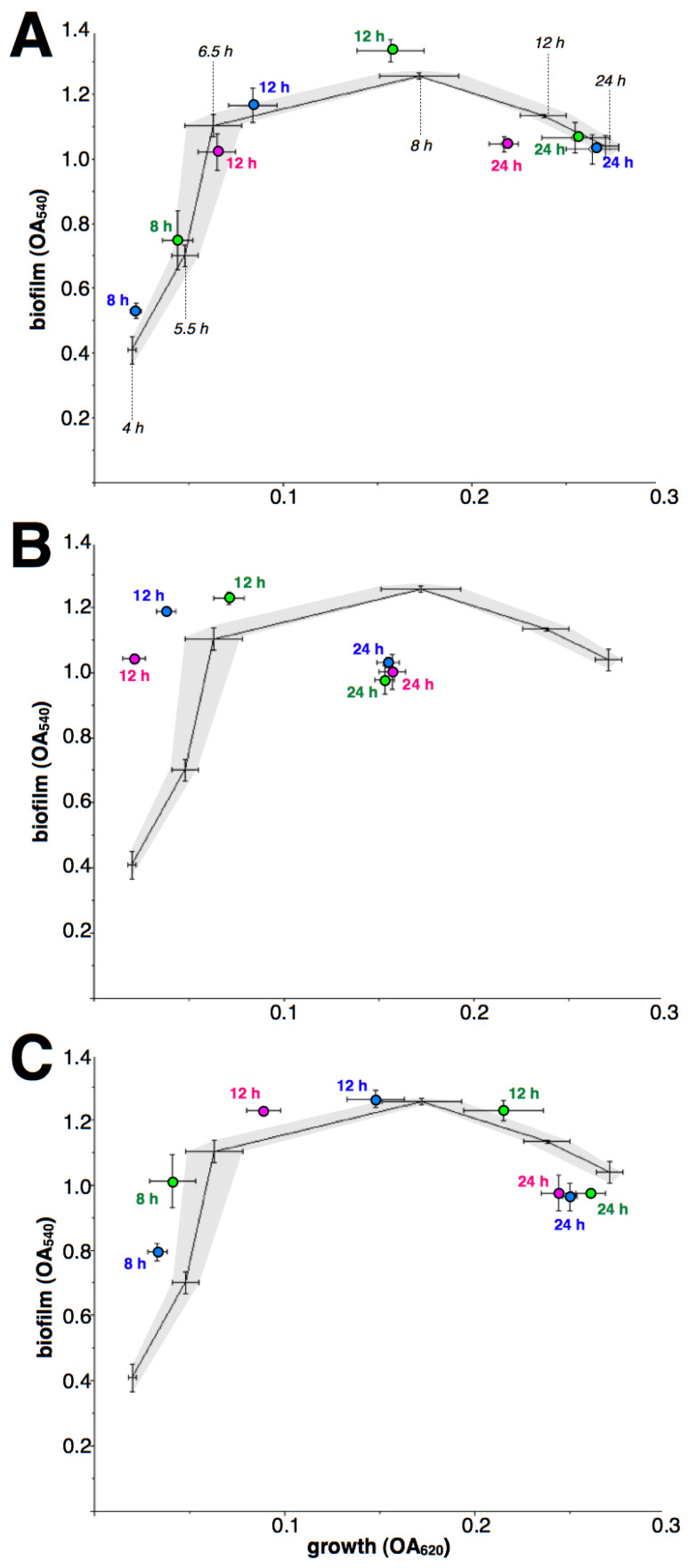
Effect of macrolides upon biofilm formation by *S. aureus* strain i1 (inducible MLS_B_). Lines (means of four independent experiments) and dashed areas (defined by standard deviations of each point) show the relationship growth/biofilm formation (as OA at 620 and 540 nm, respectively) in the absence of antibiotics at 4, 5.5, 6.5, 8, 12 and 24 h (indicated with black italics only in the top figure). The effect of ERY (**A**), CLA (**B**) and AZI (**C**) at 8, 12 and 24 h is shown as circles (means with standard deviations of four independent experiments), green for 0.5 µg/mL, blue for 1 µg/mL and magenta for 2 µg/mL; close to each symbol, numbers indicate incubation time. Missing data was caused by non-detectable growth (ERY 2/8, CLA 0.5/8, CLA 1/8, CLA 2/8 and AZI 2/8).

**Figure 4:**
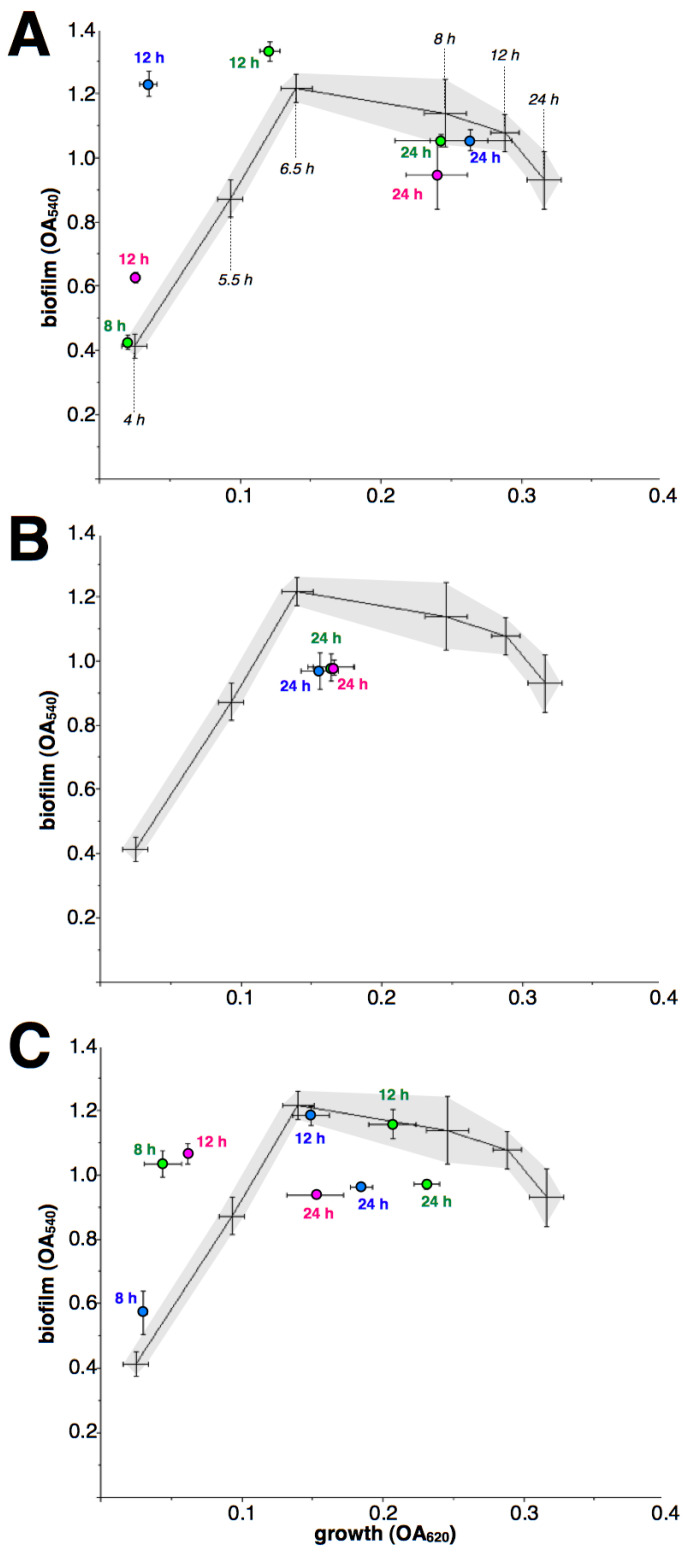
Effect of macrolides upon biofilm formation by *S. aureus* strain i2 (inducible MLS_B_). Lines (means of four independent experiments) and dashed areas (defined by standard deviations of each point) show the relationship growth/biofilm formation (as OA at 620 and 540 nm, respectively) in the absence of antibiotics at 4, 5.5, 6.5, 8, 12 and 24 h (indicated with black italics only in the top figure). The effect of ERY (**A**), CLA (**B**) and AZI (**C**) at 8, 12 and 24 h is shown as circles (means with standard deviations of four independent experiments), green for 0.5 µg/mL, blue for 1 µg/mL and magenta for 2 µg/mL; close to each symbol, numbers indicate incubation time. Missing data was caused by non-detectable growth (ERY 2/8, ERY 1/8, CLA 8 and 12 h and AZI 2/8).

As macrolides retain their inhibitory effect during the early growth of iMLS_B_ strains, a comparison against antibiotic-free controls at each incubation time is not possible. Therefore, the dynamic of biofilm formation related to cell growth is shown (Figure 3 and Figure 4), along with the results of macrolide treatment, as biofilm/growth relations. In addition, 4-h treatments were not included as they resulted in non-detectable growth. Although again results differed between drugs and strains, the most common pattern was an apparent rapid rise in biofilm formation when under macrolide treatment, at 8- and/or 12-h incubation; followed by a premature decay of biofilms after 24-h incubation.

### 2.3. Effect of Macrolides upon Biofilm Formation in Efflux-Mediated Resistant Strain

**Figure 5:**
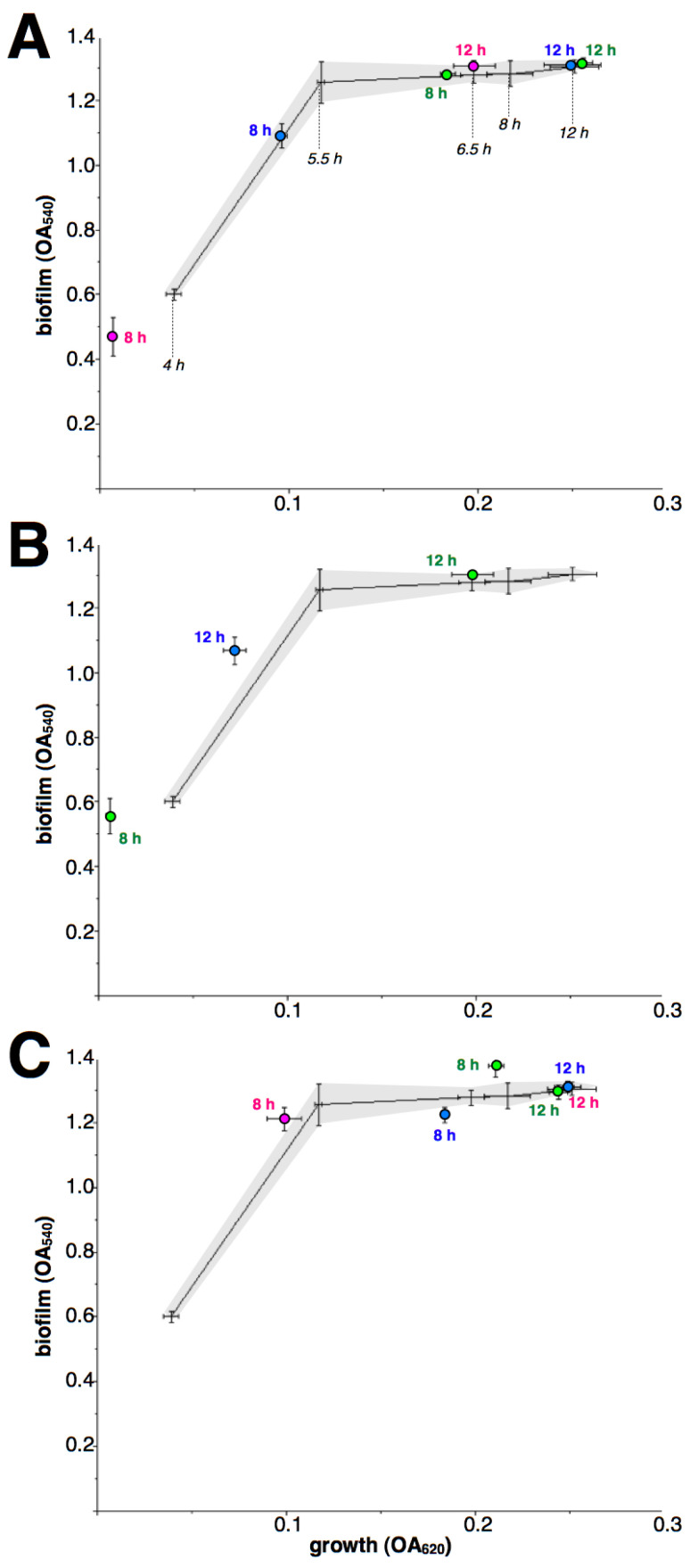
Effect of macrolides upon biofilm formation by *S. aureus* strain e1 (efflux). Lines (means of four independent experiments) and dashed areas (defined by standard deviations of each point) show the relationship growth/biofilm formation (as OA at 620 and 540 nm, respectively) in the absence of antibiotics at 4, 5.5, 6.5, 8 and 12 h (indicated with black italics only in the top figure). The effect of ERY (**A**), CLA (**B**) and AZI (**C**) at 8 and 12 h is shown as circles (means with standard deviations of four independent experiments), green for 0.5 µg/mL, blue for 1 µg/mL, and magenta for 2 µg/mL; close to each symbol, numbers indicate incubation time. Missing data was caused by non-detectable growth (CLA 2/8 and CLA 1/8).

While efflux pumps are of constitutive expression, the presence of macrolides at the tested concentrations still reduced the growth rate of the efflux isolate; hence direct comparison of biofilm formation between treated and non-treated cultures was also not possible, and dynamic comparisons are shown (Figure 5). ERY and AZI do not seem to have any effect on biofilm formation; the few time/concentrations of CLA that resulted in detectable growth did not show a significant effect either.

## 3. Discussion

Macrolide antibiotics are known to affect biofilm formation in several bacterial species; most reports on organisms that are intrinsically resistant to the drugs point to an anti-biofilm effect at clinically-relevant concentrations. However, there is little information on the effect upon biofilm formation among the Gram-positives, which are typically susceptible to macrolides. Some studies explore the effect of sub-inhibitory concentrations upon susceptible strains; for instance, at 1/8×MIC (0.003–0.005 µg/mL), ERY-induced biofilm formation in *Corynebacterium diphtheriae* [14]. Such concentrations would occur briefly, early or at the end of ERY treatment. Other papers report the effect of macrolides at clinically unattainable concentrations; e.g., a significant drop in biofilm formation by resistant *S. aureus* was observed after 24-h incubation in the presence of CLA at >32 µg/mL [10].

This study explored the effects of macrolides at clinically-relevant concentrations, i.e., around reported C_max_ values after oral administration of typical doses; it also aimed to ascertain the effects upon early biofilm development and not on late-stage, “mature” biofilms. Finally, it included strains representing each of the three most common macrolide resistance mechanisms: inducible or constitutive target modification and efflux. With the obvious limitation of using an in vitro biofilm model (i.e., attachment to a plastic surface during static incubation in liquid media), the aim was to reproduce the clinical conditions that a macrolide-resistant strain of *S. aureus* could face in a patient treated empirically with macrolides. Results show a differential effect depending on the drug, concentration, incubation time, and resistance mechanism; however, overall, macrolides seem to induce the formation of biofilm by resistant *S. aureus* isolates, except when efflux is the mechanism of resistance. These are results from a small number of clinical isolates using a single biofilm model, hence, no definitive conclusion can be drawn on said effects nor on the potential mechanisms. Further exploration is needed to fully understand this issue.

Strains expressing resistance constitutively (RN11 and c2) were the easiest to study: with continuous modification of ribosomal target by the action of methylases, these strains did withstand the macrolide concentrations from the beginning of incubation. ERY showed a consistent, biofilm-inducing effect across concentrations and times, while CLA had a similar but more modest effect and differed between both tested strains. AZI had only a significant inducing effect at the beginning (4-h incubation), but not at later stages, with even two cases of a significant reduction in biofilm formation at the lowest concentration tested (0.5 µg/mL). Strain RN11 produced a more robust and stable biofilm than strain c2 and displayed a more consistent induction of biofilm formation by macrolides, especially ERY and CLA. This suggests that macrolides induce biofilm formation the most in strong biofilm-forming strains; of course, it would be necessary to test more isolates to ascertain if this is the case. If only taking results from 24-h incubation, AZI would also seem to inhibit biofilm formation; but this is not the case during the early stages of biofilm development.

The two strains with inducible resistance produced biofilms of similar mass in the absence of macrolides (with top OA_540_ around 1.2) but with a different dynamic: while strain i1 had a slower cell growth but strong early biofilm formation and then a stabler biofilm, strain i2 grew faster but with less cell growth trapped in biofilm, which also decayed faster. In all cases, the presence of macrolides inhibited early growth, so that the effect upon biofilms could not be measured by comparing with macrolide-free controls of equal incubation times. However, by comparing each biofilm/growth pair values to the biofilm-formation dynamic of macrolide-free controls, the effect of macrolides could be ascertained. In strain i1, CLA and, to a lesser extent, AZI, increased the early formation of biofilm and also induced its decay even before growth reached the stationary phase. In strain i2, ERY and AZI also increased early biofilm formation, with AZI inducing early decay (CLA had a much strongly growth-inhibitory effect upon this strain so that only data from 24-h cultures was obtained that also suggested a rapid biofilm decay). These results indicate, again, that the effect of macrolides upon biofilm formation depends strongly on the ability and dynamic of biofilm formation of each strain. In this regard, it is important to highlight that macrolide-resistant *S. aureus* isolates produce less biofilm than susceptible ones [13]. In experiments using *S. aureus* ATCC 25923 as the control, it did have the largest biofilm formation in the absence of macrolides. On the other hand, the biofilm decay seemed to be caused by macrolides in 24-h incubation in these experiments and could be erroneously interpreted as biofilm inhibition if only late-stage biofilms are measured. The decay of staphylococcal biofilms in time has been previously reported, although not at the same time intervals: DiCicco et al., working with *S. pseudintermedius*, observed a peak at 16 h, with significant decay at 24 h (while CLA treatment had no significant impact on biofilm formation [15]). In any case, the decay could be an artifact of this in vitro model: it seems unlikely that biofilms established during an infectious process would experience decay.

Finally, and somehow to be expected, the strain with efflux-mediated resistance showed a mostly negligible effect of macrolides upon biofilm formation. While macrolides still inhibited the growth of this strain, biofilm/growth data were very similar to the macrolide-free control. A diminished intracellular concentration of macrolides due to efflux seems to eliminate the non-ribosomal effects of the drugs, which was not the case when target modification was the resistance mechanism.

Overall, these results point to a biofilm-inducing effect of macrolides on macrolide-resistant *S. aureus*, especially during early growth. Strains producing more biofilm and/or producing it faster, seem to be more affected. Exploring the effect of macrolides upon late-stage biofilm development could miss the inducing effect and perhaps even consider it an inhibitory effect, as macrolides appear to induce premature biofilm decay. While all macrolides affect biofilm formation at different times and/or concentrations, AZI seems to be the drug with the least effect. If these in vitro results correlate to effects in vivo, during a staphylococcal infection caused by macrolide-resistant strains, the empirical administration of macrolides could not only result in clinical failure due to resistance, but even in the further induction of biofilm formation. This could, in turn, complicate treatment further, as biofilms are more difficult to eradicate than planktonic bacteria. This possibility must be explored in depth to reduce the potential risk implied.

## 4. Material and Methods

### 4.1. S. aureus Strains

Clinical isolates of *S. aureus* obtained from respiratory and blood samples and identified by standard biochemical methods were kept under liquid nitrogen in glycerol-containing media and were screened for macrolide resistance using disk diffusion technique (with erythromycin disks, BBL) on Mueller–Hinton agar (Fluka) plates, following CLSI recommendations [16]. Those deemed resistant to macrolides were then tested using the D-test, with erythromycin and clindamycin disks [17] to distinguish constitutive and inducible resistance to macrolides, licosamides and group B streptogramins (cMLS_B_ and iMLS_B_, respectively), and efflux resistance phenotypes, conferring resistance to erythromycin but not clindamycin. One cMLSB (named c2), two iMLSB (named i1 and i2) and one efflux-mediated resistant (named e1) strains were selected; strain RN11 (a kind gift from R. Novick), carrying plasmid pI258 containing a constitutively-expressed *ermB* gene [18] was also included as a control. *S. aureus* strain ATCC 25923 was used as a control for antibiotic activity and biofilm assay reproducibility.

### 4.2. Biofilm Assays

Quantification of formed biofilm was made by the method of O’Toole et al. [19], growing an initial inocula of ~10^4^ cells in 100 µL brain-heart infusion (BHI, Fluka) in 96-well, flat-bottom microplates (Costar), for 4, 5.5, 6.5, 8, 12 and 24 h, at 35 °C, in the presence of erythromycin (ERY, Sigma), clarithromycin (CLA, Sigma) or azithromycin (AZI, Sigma) at final concentrations of 0.5, 1.0 or 2.0 µg/mL. After incubation, growth was measured turbidimetrically at 620 nm using a Titertek Multiskan plate reader, and then media with suspended bacteria were removed, wells were washed with water, and 0.1% crystal violet solution was added for 15 min. After removing the dye solution and washing with water, the attached dye was solubilized with 95% ethanol and brief vortexing, and the OD at 540 nm was determined. All experiments were repeated independently, four times.

### 4.3. Statistical Analysis

Results were analyzed using a one-tailed Student *t*-test performed manually.
